# Improved nutrition in adolescents and young adults after childhood cancer - INAYA study

**DOI:** 10.1186/s12885-016-2896-7

**Published:** 2016-11-08

**Authors:** J. Quidde, J. von Grundherr, B. Koch, C. Bokemeyer, G. Escherich, L. Valentini, D. Buchholz, G. Schilling, A. Stein

**Affiliations:** 1 Department of Oncology, Hematology, BMT with Section Pneumology, Hubertus Wald Tumour Center - University Cancer Center Hamburg, University Medical Center Hamburg-Eppendorf, Martinistr. 52, 20246 Hamburg, Germany; 2 Hochschule Neubrandenburg - University of Applied Sciences, Fachbereich Agrarwirtschaft und Lebensmittelwissenschaften, Brodaer Straße 2, 17033 Neubrandenburg, Germany; 3 Department of Paediatric Hematology and Oncology, University Hospital Hamburg-Eppendorf, Martinistr. 52, 20246 Hamburg, Germany; 4 Hamburger Krebsgesellschaft e.V., Butenfeld 18, 22529 Hamburg, Germany

## Abstract

**Background:**

Multimodality treatment improves the chance of survival but increases the risk for long-term side effects in young cancer survivors, so-called” Adolescents and Young Adults“(AYAs). Compared to the general population AYAs have a 5 to 15-fold increased risk of cardiovascular morbidity. Thus, improving modifiable lifestyle risk factors is of particular importance.

**Methods:**

The INAYA trial included AYAs between 18 and 39 years receiving an intensified individual nutrition counseling at four time points in a 3-month period based on a 3-day dietary record. At week 0 and 12 AYAs got a face-to-face counseling, at week 2 and 6 by telephone. Primary endpoint was change in nutritional behavior measured by Healthy Eating Index - European Prospective Investigation into Cancer and Nutrition (HEI-EPIC).

**Results:**

Twenty-three AYAs (11 female, 12 male, median age 20 years (range 19–23 years), median BMI: 21.4 kg/m^2^ (range: 19.7–23.9 kg/m^2^) after completion of cancer treatment for sarcoma (*n* = 2), carcinoma (*n* = 2), blastoma (*n* = 1), hodgkin lymphoma (*n* = 12), or leukemia (*n* = 6) were included (median time between diagnosis and study inclusion was 44 month).

The primary endpoint was met, with an improvement of 20 points in HEI-EPIC score in 52.2 % (*n* = 12) of AYAs. At baseline, median HEI-EPIC score was 47.0 points (range from 40.0 to 55.0 points) and a good, moderate and bad nutritional intake was seen in 4.3, 73.9 and 21.7 % of AYAs. At week 12, median HEI-EPIC improved significantly to 65.0 points (range from 55.0 to 76.0 points) (*p* ≤ 0.001) and a good, moderate and bad nutritional intake was seen in 47.8, 52.2 and 0 % of AYAs. No change was seen in quality of life, waist-hip ratio and blood pressure.

**Conclusion:**

Intensified nutrition counseling is feasible and seem to improve nutritional behavior of AYAs. Further studies will be required to demonstrate long-term sustainability and confirm the results in a randomized design in larger cohorts.

**Trial registration:**

Clinical trial identifier DRKS00009883 on DRKS

**Electronic supplementary material:**

The online version of this article (doi:10.1186/s12885-016-2896-7) contains supplementary material, which is available to authorized users.

## Background

Multimodality treatment, combining systemic and local therapies, offer an increasing number of patients the chance long-term survival or even cure. Currently, the number of cancer survivors is estimated to be about 15 million in the US rising to about 20 million within the next decade [1].

While improving treatment results and outcome on the one side, multimodality treatment may also increase the risk for physiological, psychological and social long-term sequelae on the other side. Adolescents and young adults (AYAs) receiving their cancer treatment during childhood are at particular risk for long-term side effects. According to the Childhood Cancer Survivor study (CCSS, *n* = 10,397) 2 out of 3 AYAs have treatment related long-term toxicities [2].

The major long-term toxicities and cause of mortality after treatment of childhood cancer are cardiovascular diseases like cardiomyopathy, chronic heart failure (CHF) and valvular problems [3]. Compared to general population AYAs have a 5 to 15-fold increased risk of cardiovascular morbidity [3–5]. The individual risk is determined by treatment related factors (e.g. type of chemotherapeutic agents, application schedule, number of cycles, cumulative dose of different agents, and combination with radiotherapy) and non-treatment related factors (nicotine abuse, diabetes mellitus, dyslipoproteinemia and hypertension [6]. AYAs who have received anthracycline-based treatment combined with chest radiation possess the highest risk for cardiovascular late toxicity, which may further be enhanced by non-treatment related factors [6].

Whereas large clinical trials are conducted to improve patient outcome, both in terms of cure rates and reduction of long-term toxicities, prospective studies evaluating modifications of lifestyle factors as potential non-treatment related risk factors for long-term toxicity in AYAs are lacking.

In adult survivors of breast and prostate cancer some studies have demonstrated that physical activity and healthy eating can increase survival [7, 8].

The WHEL study showed that telephone nutrition counseling can achieve major increases in the intake of micronutrient- and phytochemical-rich vegetables, fruit and fiber in breast cancer survivors [9]. The ENRGY trial has shown that a behavioral weight loss intervention can lead to clinically meaningful weight loss in overweight/obese breast cancer survivors [10]. However, with a median age at diagnosis of more than 60 years for these cancer survivors, the psychosocial background and living conditions largely differ from AYAs. Little is known about the feasibility and the effectiveness of a lifestyle intervention in an AYA population at risk for cardiovascular disease.

Furthermore, only 10 % of the cancer survivors are following a healthy lifestyle without any additional intervention [11]. A large number of cancer survivors are overweight (58 %), eat less than 5 times per day fruits and vegetables (82 %) and perform no sport activities (55 %) [11].

The correlation between nutrition and cardiovascular disease in AYAs is unknown, but has been intensively studied in coronary heart disease (CHD) patients. For this patient group a relevant risk reduction for development of CHF by healthy nutrition was demonstrated (e.g. by 30 % with a Mediterranean diet or by 13 % following the criteria of “Dietary Approaches to Stop Hypertension”) [12–14]. Both diets are rich of fibers, fruits and vegetables, which mirrors the nutrition recommendations of the “Deutsche Gesellschaft für Ernährung” (DGE) (see Additional file 1: Table S1 for recommendations of the DGE) [15]. In CHD patients an intensive, individualized nutrition counseling leads to an improvement of nutritional status and behavior and finally quality of life [13].

The INAYA trial reported here was performed to evaluate the feasibility and the impact of an intensified nutrition counseling in the particularly at risk population of AYAs.

## Methods

### Trial eligibility

AYAs aged from 18 to 39 years with at least one treatment related (e.g. anthracycline based chemotherapy or chest radiation) or at least one non-treatment related (nicotine abuse, diabetes mellitus, dyslipoproteinemia or hypertension) risk factor for cardiovascular disease were eligible. All AYAs had completed cancer treatment with curative intent, were currently considered in remission and were receiving aftercare within our multidisciplinary survivorship clinic.

The trial was approved by the institutional review board and registered (Clinical trial identifier DRKS00009883 on DRKS). All patients provided written informed consent before study entry.

### Study design

Prior to baseline assessment and after 12 weeks the AYAs filled in a 3-day dietary record (“Freiburger Ernährungsprotokoll”) providing data to calculate the “Healthy Eating Index- European Prospective Investigation into Cancer and Nutrition” (HEI-EPIC) [16].

**Fig. 1:**
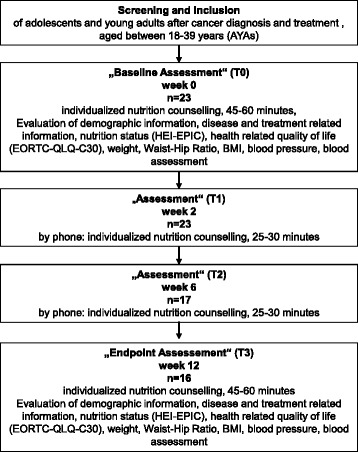
Study design

At baseline and after 12 weeks all AYAs got an intensified face-to-face nutrition counseling of 60 min performed by the same registered dietitian and based on the recommendations of the DGE (see Additional file 1: Table S1) [15]. Depending on the HEI-EPIC results and reported nutrition problems, recommendations of the DGE were modified and individually tailored to the needs of every AYA. Additionally, demographic information and medical history was collected at baseline. Waist-hip ratio (WHR), body mass index (BMI), blood pressure (RR), health-related quality of life (HRQOL, measured by EORTC QLQ-C30 [17]) and laboratory parameters (AST, ALT, HbA1c, total cholesterol, HDL-cholesterol, LDL-cholesterol, C-reactive protein (CrP)) were assessed at baseline and after 12 weeks. At weeks 2 and six nutrition counseling of 30 min was repeated by telephone. (see Fig. 1 for study flowchart).

### Healthy eating index - European prospective investigation into cancer and nutrition

HEI-EPIC is an established instrument to evaluate the dietary behavior [18]. In the present pilot study the validated German version of HEI, the HEI-EPIC was used [16].

The HEI-EPIC distinguishes the following eight food groups: drinks, vegetables, fruits, cereals/potatoes, milk/dairy products, meat/sausages/fish/eggs, fats/oil and sweets/snacks. Based on a calculation described by Rüsten et al. 0–10 points for each group of food with up to 20 points for fruits, vegetables and drinks were calculated [16]. The sum score range from 0 to 110 points. A sum score ≤ 40 points indicate a bad, > 40–64 points a moderate and ≥ 65 points a good dietary behavior [16, 19].

### Statistics

The primary endpoint was the rate of AYAs with a relevant improvement in nutritional behavior measured by HEI-EPIC (increase of at least 20 points) between weeks 0 and 12. Secondary endpoints were the change in median HEI-EPIC, the assessment of HRQOL by the EORTC QLQ-C30, WHR, BMI, RR and laboratory assessment regarding dyslipoproteinemia and cholesterinemia. Pre-post differences of the secondary endpoints were analyzed with Wilcoxon test for depended samples in an exploratory fashion. In addition, retrospective BMI sub groups were evaluated and patients were classified as: underweight (≤18.49 kg/m^2^; *n* = 5), normal weight (>18.5–24.99 kg/m^2^; *n* = 14) and overweight/obese (≥ 25.0–44.99 kg/m^2^; *n* = 4). No formal comparison of BMI subgroups was performed due to small numbers.

### Sample size calculation

Based on our experience in the survivorship clinic, about 25 % of patients improve their nutritional behavior (increase in 20 points in HEI-EPIC) after a general nutrition counseling. With the intensive, individualized nutrition counseling at least 50 % of AYAs should improve their nutritional intake by 20 points on HEI-EPIC to regard the intervention as meaningful. The probability to accept the intervention as promising (improvement rate ≥50 % of AYAs), in spite of a true improvement rate of ≤25 % only, was set at 0.1 (type I error). The probability to erroneously reject the intervention as not sufficiently efficient (≤25 %), although the true improvement rate is meaningful (≥50 %) was set at 0.2 (type II error, corresponding to a power of 80 %). According to these parameters and using a standard single-stage phase II design with a one sided test and including 10 % drop outs, *n* = 21 AYAs had to be recruited [20].

## Results

### Patients’ characteristics

Twenty-three AYAs, 11 female and 12 male, were included in the INAYA study. Median age of cancer diagnosis was 16.0 years (range: 10.0–17.0 years), median age at time of study inclusion was 20.0 years (range: 19.0–23.0 years). Median time between diagnosis and study inclusion was 44 months (range: 11.0–237 months). Cancer diagnosis included sarcoma (*n* = 2), carcinoma (*n* = 2), blastoma (*n* = 1), hodgkin lymphoma (*n* = 12), or leukemia (*n* = 6).

All AYAs (100 %) presented treatment related risks factors for cardiovascular diseases that were application of anthracyclines (*n* = 22, 95.7 %), chest radiation (*n* = 9, 39.1 %) or both (*n* = 14, 60.9 %). Additional 8 (34.8 %) AYAs had non-treatment related risk factors: smoking (*n* = 5, 21.7 %), overweight with BMI > 25.0 kg/m^2^ (*n* = 4, 17.4 %) and hypertension (*n* = 1, 4.3 %), but no diabetes mellitus (*n* = 0, 0 %). One AYA had more than one non-treatment related risk factor.

Compliance rate was 100 % (*n* = 23) at baseline and week 2 but decreased to 73.9 % (*n* = 17) at week 6 and 69.6 % (*n* = 16) at week 12. Therefore, overall attrition rate was 30 %. Reasons for drop out were disease relapse (*n* = 1), lack of time due to high work load (*n* = 1), lack of time due other reasons (*n* = 3) and two were lost to follow up.

### Primary endpoint

In 52.2 % (*n* = 12) of AYAs the median HEI-EPIC score improved by more than 20 points from baseline to week 12. Thus, the primary endpoint of an improvement in nutritional behavior by more than 20 points in more than 50 % of AYAs measured by HEI-EPIC was met, demonstrating the feasibility of the intensive, individualized nutrition counseling and a high rate of improvement in nutritional behavior.

### Secondary endpoints

#### HEI-EPIC

**Fig. 2:**
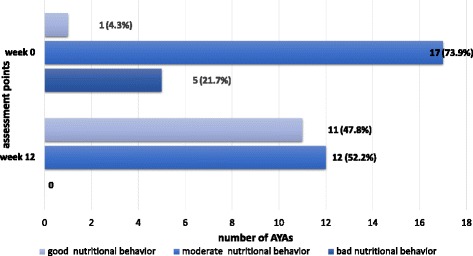
Nutritional behavior in AYAs at baseline and week 12

At baseline, the median HEI-EPIC score was 47.0 points (range: 40.0–55.0 points) representing a moderate dietary behavior. A good, moderate and bad nutritional intake was seen in 4.3, 73.9 and 21.7 % of AYAs. At week 12, median HEI-EPIC score improved significantly to 65.0 points (range: 55.0–76.0 points) (*p* ≤ 0.001) representing a good dietary behavior. Good, moderate and bad nutritional intake was seen in 47.8, 52.2 and 0 % of AYAs (Fig. 2).

Nutrition scores improved significantly in all food groups, except meat/sausages/fish/eggs/soy products and fats/oil (Table 1).

**Table 1:** Change in median nutrition intake of different HEI-EPIC food groups

Food groups	Week 0 (T0)	Week 12 (T3)	*p*-value
drinks	4.8 (3.3–9.3)	8.4 (6.9–10.1)	0.001
vegetables	1.8 (0.8–2.4)	3.1 (1.7–3.9)	0.001
fruits	1.5 (0.0–2.4)	2.4 (0.5–3.6)	0.030
cereals/cereals products/potatoes	2.3 (1.7–2.9)	2.9 (2.3–3.4)	0.038
milk/milk products/dairy products	3.3 (1.3–2.9)	1.8 (1.6–3.9)	0.041
meat/sausages/fish/eggs/soy products	1.2 (0.7–1.8)	1.5 (0.8–1.8)	0.452
fats/oil	1.6 (1.2–2.2)	2.1 (1.8–2.6)	0.178
sweets/snacks/alcohol	2.4 (1.4–2.8)	0.9 (0.6–1.8)	0.001

HEI-EPIC score numerically improved in all BMI subgroups (Table 2).

**Table 2:** HEI-EPIC of different BMI groups

BMI groups	HEI-EPIC score week 0 (T0)	HEI-EPIC score week 12 (T3)
underweight AYAs:	49.0 (38.0–57.5)	78.0 (57.0–80.5)
BMI <18.5 kg/m^2^ (*n* = 5)		
normal weight AYAs:	50.5 (45.8–56.3)	62.5 (53.3–72.3)
BMI ≥18.5–24.9 kg/m^2^ (*n* = 14)		
overweight AYAs:	37.5 (27.3–44.0)	60.0 (49.8–69.5)
BMI ≥25.0 kg/m^2^ (*n* = 4)		

#### Quality of life

For quality of life analysis we used the global health status/quality of life (GHS/QOL) score of the EORTC QLQ C-30 questionnaire. The median GHS/QOL score did not change and was 83.3 points (range: 66.7–91.7 points) at week 0 and 83.3 points (range: 66.7–91.7 points) at week 12 (*p* = 0.332) (Table 3). A clinical relevant improvement of GHS/QOL score (≥ 10 points) was seen in 21.7 % (*n* = 5) of AYAs. While normal weight patients did not improve their median GHS/QoL (83.3 at week 0 and 12), patients of under- or overweight seemed to increase the GHS/QoL score (underweight: 66.7 to 83.3; overweight 75.0 to 87.5 from week 0 to 12). Detailed results for functioning, symptom and single item scores of the EORTC QLQ C-30 questionnaire are shown in Additional file 1: Table S2.

**Table 3:** Secondary endpoints: quality of life, BMI, blood pressure and WHR

Selected secondary endpoints	Week 0 (T0)	Week 12 (T3)	*p*-value
BMI (kg/m^2^)	21.4 (19.7–23.9)	20.4 (19.0–23.9)	0.218
Quality of life (GHS/QoL points)	83.3 (66.7–91.7)	83.3 (66.7–91.7)	0.332
Blood pressure (mmHg)	110/70 (105–135/60–90)	110/75 (95–135/60–90)	0.015/0.605
Waist hip ratio (WHR)	0.80 (0.69–0.97)	0.77 (0.66–0.98)	0.349

#### Body Mass Index (BMI)

At baseline 5 AYAs were underweight, 14 AYAs normal weight and 4 AYAs overweight according to WHO criteria. After nutrition counseling at week 12, the amount of normal weight AYAs increased to 17, with only four underweight and two overweight/obese AYAs remaining.

Before nutrition counseling 5 patients claimed weight loss as one of the goals of taking part in that trial. All of them reached their aim to lose weight. The overweight/obese patients (*n* = 4) lost a median of 4.3 kg (range: 1.4–7.9 kg).

The median BMI at baseline was 21.4 kg/m^2^ (range: 19.7–23.9 kg/m^2^) and 20.4 kg/m^2^ (range: 19.0–23.9 kg/m^2^) at week 12 (*p* = 0.218) (Table 3). In underweight and normal weight patients the median BMI remained unaffected 17.7 kg/m^2^ (range: 16.8–18.0 kg/m^2^) and 21.4 kg/m^2^ (range: 20.0–23.4 kg/m^2^) at baseline and 17.7 kg/m^2^ (range: 16.8–18.2 kg/m^2^) and 20.7 kg/m^2^ (range: 20.0–23.2 kg/m^2^) at week 12, respectively. In overweight patients a slight decrease in median BMI from 29.1 kg/m^2^ (range: 25.2–40.0 kg/m^2^) to 27.3 kg/m^2^ (range: 24.6–37.8 kg/m^2^) was noted.

#### Blood pressure (RR)

During the 12 weeks intervention period the median RR remained stable with 110/70 mmHg (range: 110–125/70–80 mmHg) at baseline and 110/75 mmHg (range: 105–125/70–80 mmHg) at week 12 (Table 3).

#### Waist-hip ratio (WHR)

All patients WHR were within the normal range <0.85 for women and <1.0 for men. Median WHR did not change significantly from baseline to week 12, neither in the overall population nor in the BMI subgroups.

Median WHR was 0.80 (range: 0.72–0.80) at baseline and 0.77 (range: 0.71–0.80) at week 12 (*p* = 0.349). 65.2 % (*n* = 15) had a lower and 30.4 % (*n* = 7) had a higher WHR in week 12 (Table 3). No relevant differences in WHR changes over time were noted within the BMI subgroups.

#### Biochemical parameters (AST, ALT, HbA1c, total cholesterol, HDL-cholesterol, LDL-cholesterol, CRP)

Intensified nutrition counseling had no significant effect on biochemical parameters. Detailed results for AST and ALT, HbA1c, total-cholesterol, LDL-cholesterol, HDL-cholesterol and CRP are shown in Additional file 1: Table S3.

## Discussion

This first, prospective life-style modification trial in the particular patient group of AYAs demonstrate the feasibility of an intensified, individual nutrition counseling and results in high rates of improvement in nutritional behavior. An improvement of more than 20 points was seen in 52.2 % (*n* = 12) of the AYAs. Thus, the primary endpoint of this study was met. The median HEI-EPIC score changed significantly from a moderate (47.0 points) to a good nutritional behavior (65.0 points). Overall, the number of AYAs with good nutritional behavior was increased by the INAYA intervention from one to 11.

The AYA population relevantly differs from the elderly survivor patients and prospective trials in this distinct setting are lacking. AYAs have to deal with a variety of relevant problems including educational and occupational issues, partnership and family planning, besides lifestyle and nutrition. Of note, compared to previously reported rates of healthy food intake of up to 18 % in elderly cancer survivors without nutrition counseling, the baseline rate in our population seemed to be even lower (*n* = 1, 4.3 %) [11].

Despite the particular vulnerability of the AYA population prospective trials about nutrition counseling are lacking. The majority of published nutrition intervention trials include either non-AYA or non-cancer patients. Nevertheless, similar to the INAYA trial, a large variety of trials in different patient population could demonstrate the feasibility and efficacy of lifestyle interventions.

A review of 21 trials about nutrition counseling in primary care setting demonstrated that moderate- or high-intensity counseling interventions, including use of interactive health communication tools, can reduce the consumption of saturated fat and increase the intake of fruit and vegetable and therefore improve nutritional behavior. The patient population included was very heterogeneous and is not comparable to AYAs [21]. In breast cancer patients, a randomized interventional trial showed a significant improvement in vegetable consumption 8 weeks after nutrition counseling [22]. In regard of dietary behavior, the best-investigated patient groups are those with hypertension or coronary heart disease (CHD). In these groups structured educational programs and intensified nutrition counseling improve dietary behavior and quality of life [23].

Although survivors usually have an increased risk of poor HRQOL, the baseline GHS/QoL score was relatively high (83.3 points) in our cohort [24]. HRQOL assessment refers to a multidimensional construct, considering the subjective perceptions of disease symptoms, treatment side effects as well as physical, emotional, social and cognitive functions. Therefore, it might be difficult to improve the overall HRQOL by the improvement of nutritional behavior.

Limitations of the INAYA study are the relatively low number of participants, limiting the interpretation of results particularly of the subgroup evaluations. Furthermore, the attrition rate of 30 % limits the follow up data. The availability for phone consultations was limited, mainly due to educational or occupational engagements of our AYA patients. Therefore, for future trials the follow up procedures should be carefully evaluated. One way could be to send nutrition information and reminders by e-Mail, Apps or SMS. Another possibility could be to extend the intervals and re-counsel the AYAs at the next regular aftercare appointment. The relatively short intervention and follow up period does not allow evaluating the sustained efficacy of the INAYA protocol. Further trials will need to confirm the sustainability of the INAYA approach, although periodical re-counseling will likely be necessary to ensure long-term healthy eating. Due to the single arm design a control-group is missing and the efficacy of the INAYA approach cannot be demonstrated. However, a high rate of 50 % of AYAs with an improvement in nutritional behavior was noted, clearly pointing towards further evaluation of the INAYA approach in a randomized setting.

## Conclusion

Intensified nutrition counseling is feasible and seem to improve short-term dietary behavior of AYAs and thus potentially reduces the long-term risk for developing cardiovascular diseases. Thus, intensified lifestyle modification approaches flanked by long-term follow up are required in AYAs.

## Additional file

Additional file 1: Table S1. 10 guidelines of the German Nutrition Society (DGE) for a wholesome diet. **Table S2**. Results of EORTC QLQ C-30 questionnaire. **Table S3**. Change in blood values ASAT, ALAT, HbA1c, Total cholesterol, LDL-cholesterol, HDL-cholesterol and CrP. (DOCX 17 kb)

## Abbreviations

ALT: Alanine aminotransferase
AST: Aspartate aminotransferase
AYA: Adolescents and young adults
BMI: Body mass index
CCSS: Childhood cancer survivor study
CHD: Chronic heart disease
CHF: Chronic heart failure
CRP: C-reactive protein
DGE: “Deutsche gesellschaft für ernährung”
DRKS: Deutsches register klinischer studien
e.g.: Exempli gratia
EORTC QLQ-C30: European Organisation for Research and Treatment of Cancer Qualitiy of Life Core Questionnaire 30
GHS/QOL: Global health status/quality of life
HbA1c: Hemoglobin A1c
HDL-cholesterol: High-density lipoprotein cholesterol
HEI-EPIC: Healthy eating index - European prospective investigation into cancer and nutrition
HRQOL: Health-related quality of life
INAYA: Improved nutrition in AYAs
LDL-cholesterol: Low-density lipoprotein cholesterol
RR: Blood pressure
S: Supplement
WHR: Waist-hip ratio
