# Supplementary material for: Improved nutrition in adolescents and young adults after childhood cancer - INAYA study
Source: BMC Cancer. 2016 Nov 8;16:872. doi: 10.1186/s12885-016-2896-7 (PMC5101649; doi:10.1186/s12885-016-2896-7)
Supplement: Additional file 1: Table S1. — 10 guidelines of the German Nutrition Society (DGE) for a wholesome diet. Table S2. Results of EORTC QLQ C-30 questionnaire. Table S3. Change in blood values ASAT, ALAT, HbA1c, Total cholesterol, LDL-cholesterol, HDL-cholesterol and CrP. (DOCX 17 kb) [file 12885_2016_2896_MOESM1_ESM.docx]

## ***Supplement 1:*** 10 guidelines of the German Nutrition Society (DGE) for a wholesome diet

| 10 guidelines of the DGE |  |
| --- | --- |
| **Enjoy the diversity of foods available** | A wholesome diet includes a variable choice, adequate quantities and an appropriate combination of high-nutrient and low-energy food. Choose mainly plant-based foods. They have a health-promoting effect and foster a sustainable diet |
| **Ample cereal products and potatoes** | Bread, grain flakes, pasta, rice, preferably from whole grain, and potatoes contain plenty of vitamins, minerals and dietary fibre as well as phytochemicals. Consume these foods preferably with low-fat ingredients. At least 30 grams of dietary fibre daily, especially from whole-grain products, are recommended. A high intake lowers the risk of various nutrition-related diseases |
| **Fruit and vegetables - take ‘5 a day’** | Enjoy 5 portions of fruit and vegetables daily, as fresh as possible, cook for a short time only, or occasionally, take 1 serving as a juice or smoothie – ideally with each main meal and also as a snack between meals: You profit by consuming plenty of vitamins, minerals, dietary fibre and phytochemicals and lower the risk of nutrition-related diseases. Rather favour seasonal products |
| **Milk and dairy products daily; fish once to twice a week; meat, sausages and eggs in moderation** | These foods contain valuable nutrients, e.g. calcium in milk, iodine, selenium and n-3 fatty acids in saltwater fish. Choose fish products from recognised sustainable sources. As part of a wholesome diet, you should not eat more than 300 - 600 grams of meat and sausages per week. Meat contains minerals and vitamins B1, B6 and B12. From the health point of view, white meat (poultry) is more favourable than red meat (beef, pork). Rather choose low-fat products, especially with meat and dairy products |
| Fat and fatty foods in moderation | Fat provides essential fatty acids and foods containing fat also comprise fat-soluble vitamins. Fat is particularly high in energy, therefore an increased intake of dietary fat can promote overweight. Too many saturated fatty acids increase the risk of dyslipidemia with the possible consequence of cardiovascular diseases. Rather favour vegetable oils and fats (e.g. canola oil, soybean oil and margarines produced therefrom). Be aware of hidden fat found in several meat and dairy products, pastry, sweets, fast food and convenience products. Overall, 60 - 80 grams of fat daily is sufficient. |
| Sugar and salt in moderation | Only occasionally consume sugar and food or beverages containing various kinds of sugar (e.g. glucose syrup). Be creative in flavouring with herbs and spices, but use little salt. Rather favour iodised and fluoridated table salt. |
| Plenty of fluid | Water is essential to life. Make sure your daily fluid intake is approximately 1½ litres. Rather choose water, carbonated or non-carbonated, and other beverages low in calories. Only rarely drink sugar sweetened beverages. They are high in energy, therefore an increased intake can promote overweight. Consume alcoholic drinks only occasionally and only in small amounts due to the health risks associated with them. |
| Prepare carefully cooked dishes | Preferably cook foods on low heat, if possible for a short time, using little amount of water and fat. This will preserve the natural taste, conserve the nutrients and avoid the formation of harmful substances in food. Use fresh ingredients whenever possible. This helps to reduce unnecessary packaging waste. |
| Take your time and enjoy eating | Take a break while you eat and do not eat in passing. Allow plenty of time for eating, this promotes your sense of satiation. |
| Watch your weight and stay active | Combine a wholesome diet along with plenty of physical exercise and sport (30 – 60 minutes daily). This will help you to control your weight. For example, you can walk or take the bicycle from time to time. This protects the environment and promotes your health. |

***Supplement 2*** *Results of EORTC QLQ C-30 questionnaire*

| Quality of life | week 0  (T0) | week 12  (T3) | p-value |
| --- | --- | --- | --- |
| Physical Function | 93.3 (60.0 – 100) | 100 (66.6 – 100) | **0.039** |
| Role Function | 83.3 (33.3 – 100) | 100 (33.3 – 100) | 0.189 |
| Emotional Function | 75.0 (41.7 – 100) | 75.0 (8.3 -100) | 0.380 |
| Cognitive Function | 66.7 (16.7 – 100) | 83.3 (33.3 – 100) | 0.205 |
| Social Function | 100 (50 – 100) | 100 (50 – 100) | 0.498 |
| Fatigue | 33.3 (0 – 88.9) | 22.2 (0 – 100) | 0.170 |
| Nausea/Vomiting | 0.0 (0.0 – 100) | 0.0 (0.0 – 66.7) | 0.400 |
| Pain | 16.7 (0.0 – 66.7) | 0.0 (0.0 – 66.7) | 0.524 |
| Dyspnoea | 0.0 (0.0 – 100) | 0.0 (0.0 – 66.7) | 1.0 |
| Insomnia | 0.0 (0.0 – 100) | 0.0 (0.0 – 100) | 0.123 |
| Appetite loss | 0.0 (0.0 – 100) | 0.0 (0.0 – 66.7) | 0.157 |
| Constipation | 0.0 (0.0 – 66.7) | 0.0 (0.0 – 33.3 | 0.357 |
| Diarrhoea | 0.0 (0.0 – 100) | 0.0 (0.0 – 100) | 0.365 |
| Financial problems | 0.0 (0.0 – 66.67) | 0.0 (0.0 – 100) | 0.888 |

***Supplement 3*** *Change in blood values ASAT, ALAT, HbA1c, Total cholesterol, LDL-cholesterol, HDL-cholesterol and CrP*

| biochemical parameters | week 0 (T0) | week 12 (T3) | p-value |
| --- | --- | --- | --- |
| ASAT (U/I) | 22.0 (18.0 – 27.0) | 20.0 (17.0 – 23.0) | 0.123 |
| ALAT (U/I) | 22.0 (19.0 – 32.0) | 24.0 (21.0 – 30.0) | 0.098 |
| HbA1c (%) | 4.9 (4.6 – 5.1) | 5.0 (4.8 – 5.2) | 0.190 |
| Total cholesterol (mg/dl) | 163.0 (147.0 – 180.0) | 162.0 (146.0 – 180.0) | 0.570 |
| LDL cholesterol (mg/dl) | 85.0 (73.0 – 102.0) | 92.0 (71.0 – 102.0) | 0.629 |
| HDL cholesterol (mg/dl) | 57.0 (44.0 – 69.0) | 54.0 (48.0 – 66-0) | 0.864 |
| CrP (mg/l) | 0.0 (0.0 – 0.0) | 0.0 (0.0 – 0.0) | 0.285 |
